# BK virus‐associated urothelial carcinoma—A supra‐regional cohort of kidney transplant recipients

**DOI:** 10.1002/bco2.70183

**Published:** 2026-03-12

**Authors:** Noah Beetge, Luis Ribeiro, Rhana Zakri, Catherine Horsfield, Jonathon Olsburgh

**Affiliations:** ^1^ GKT School of Medical Education London UK; ^2^ Guy's and St Thomas' NHS Foundation Trust London UK

**Keywords:** BK virus, BK virus‐associated nephropathy, kidney transplant recipients, SV40 large T antigen, urothelial carcinoma

## Abstract

**Objectives:**

This study aims to study the clinical features, pathological findings, and outcomes of BK virus‐associated urothelial carcinoma (UC) in kidney transplant recipients (KTRs).

**Patients and Methods:**

The study was conducted in a retrospective cohort of KTRs with histologically confirmed UC managed at a UK supra‐regional transplant urology centre from November 2006 to January 2026. BK virus status was determined using polymerase chain reaction (PCR) testing, and tumour samples were assessed for SV40 large T antigen. Patients were stratified by BKV and SV40 status to enable comparison of tumour grade, stage, and other clinicopathological features.

**Results:**

24 KTRs met the inclusion criteria; 11 of whom were BKV+. The mean follow‐up time post‐UC diagnosis was 7.3± 6.1 years. Mean age at UC diagnosis was 59.9 ± 14.6 years. 90.9% of BKV+ patients had high‐risk UC compared to 30.8% of the BKV− group (*p* = 0.005). Tumour grade at diagnosis was higher in BKV+ patients (*p* = 0.013). SV40 Large T antigen was detected in 33.3% of cases, all with a previous history of BKV infection. These tumours were all high grade (G3) and had tended to be a higher stage at diagnosis than SV40− tumours (*p* = 0.050). UC in BKV+ patients was not diagnosed earlier post‐transplant (*p* = 0.616). There was no difference in survival probability between the two cohorts (*p* = 0.639).

**Conclusion:**

BKV infection in KTRs was associated with aggressive, high‐grade UC. Screening and timely adjustment of immunosuppression are essential to protect this at‐risk population. Further studies are required to clarify the oncogenic potential of BKV and optimise management strategies.

## 1 INTRODUCTION

The KTR population has been shown to have a 1.4–4.8 times higher risk of developing UC compared to the general population.^1^, ^2^ Furthermore, this population has a 2.5‐fold higher mortality rate from bladder cancer, which may be due to the significantly higher progression rates of UC in the bladder.^3^ Risk factors for UC in KTRs include age, immunosuppression, cyclophosphamide, smoking, aristolochic acid and viruses, including BKV.^4^, ^5^

BKV is part of the Polyomaviridae family: small, icosahedral, non‐enveloped viruses that infect mammalian, bird and fish species.^6^ The BKV genome consists of three distinct regions: an early coding region for large T and small t antigens, a noncoding region and a late coding region.^7^ There is a seroprevalence of 60%–90% in the adult population worldwide.^8^ Initial infection occurs mostly in children, with a median age of 4–5 years.^9^ Following primary infection, the virus establishes latency in the genitourinary tract, where it remains inactive with minimal episomal replication in healthy individuals.^10^

In immunosuppressed KTRs, BKV commonly reactivates in the genitourinary tract. The major risk factor associated with increased viral replication is the intensity and type of immunosuppressive regimens, particularly tacrolimus and/or steroid‐based regimens. The infection occurs in the chronological order of viruria, viremia and BK virus‐associated nephropathy (BKVN). Reactivation occurs most frequently in the first year post‐transplant, with 39.5% of individuals developing BKV viruria within this period.^11^ This is a significant risk factor for BKVN which occurs in 10%–13% of KTRs.^12^, ^13^ BKVN can cause a decline in kidney function, and patients with BKVN face a 4.4‐fold higher risk of graft loss.^14^

Previous research suggests a causal relationship between BKV reactivation and urinary tract cancers. Patients with BKVN had 6.46‐fold higher rates of urinary tract cancer.^15^ Particularly invasive bladder carcinoma was associated with a standardised incidence ratio (SIR) of 4.5 in KTRs who had received treatment for BKVN as opposed to those who had not.^16^ In KTRs with BKVN, 30% of the UC expressed BKV LTag.^17^ The mechanisms around BKV's carcinogenic impact have been elucidated from in vitro and in vivo studies. BKV causes TAg‐mediated disruption of the tumour suppressor genes p53 and pRb, resulting in cell‐cycle dysregulation and apoptosis.^18^ Iwasaki et al. reported 40 cases of BKV‐associated UC since 2002; of those tested for p53 (*n* = 28), 92.9% showed diffuse positivity.^17^

This study aims to investigate the association between BKV infection and the clinical features, pathological findings and outcomes of UC in our KTR population. We compared the age at diagnosis, time post‐transplantation and UC pathology characteristics between BKV+ and BKV− patients. Furthermore, this study assessed the influence of BKV status on patient survival and identified potential risk factors.

## 2 PATIENTS AND METHODS

### 2.1 Data collection

This is a retrospective cohort study conducted at a UK supra‐regional transplant urology centre between November 2006 and January 2026. Records of newly diagnosed UC in kidney transplant recipients (KTRs) were systematically collected from the inception of the Transplant Urology service in November 2006. During this study period, 3459 KTs were performed.

Some patients in our cohort had undergone transplantation prior to November 2006. From 1975, the first transplant from our series, up to November 2006, a further 4966 transplants were carried out. It is not possible to determine how many of these patients subsequently developed UC prior to systematic data collection, as relevant records are unavailable. The total transplant figures include SPK transplants, but not SLK transplants or pediatric transplants, of which there were none in our series.

KTRs with pathologically confirmed UC and available clinical and pathology data were included. Patients were excluded if they had UC prior to transplantation, if the bladder cancer was non‐urothelial, or if medical data were unavailable. Additionally, if BK reactivation, in the form of viremia or viruria, had not been tested for, they were noted but not included in the analysis.

This study is part of a GSTT service evaluation clinical audit titled: Service Evaluation of Transplant Viruses and Urological Conditions including Cancer (IRB no. 17458).

### 2.2 Definitions

Patient data were extracted from electronic health records and the Transplant Database, and anonymised data were synthesised using Excel (Microsoft), including patient characteristics: age, gender, cause of ESRD and smoking status; transplant history including type, date, retransplant and donor characteristics: gender, cause of death, age and biological age of donor kidney at UC diagnosis; tumour characteristics: date of UC diagnosis, location, stage, grade, histological subtype and details on metastases. BK virus status was analysed using viral load, earliest date of detection, biopsy proven BKVN and peak viremia. Data regarding treatment, outcomes, including survival, and immunosuppression regimens were collected.

BKV infection status was assessed using real‐time polymerase chain reaction (PCR) assay of BKV DNA in whole blood or urine. A positive value at any time post‐transplant was considered evidence of BKV infection. Decoy cells alone were not sufficient evidence without further testing. BK virus in tumour samples was analysed with immunohistochemistry directed against cross‐reacting SV40 large T‐antigen.

### 2.3 Statistical analysis

Urothelial carcinoma diagnoses were confirmed through cystoscopy with biopsy, followed by histopathological examination. Tissue samples were evaluated, and tumours were classified according to the TNM staging system.^19^

Patients were categorised into either BKV‐positive (BKV+) or BKV‐negative (BKV−). Statistical analysis was performed using SPSS.^20^ For patient characteristics, descriptive statistics (mean, median, standard deviation and percentage) were used. For categorical variables, chi‐squared testing or Fisher's exact test was used. Normality was assessed using the Kolmogorov–Smirnov test and the Shapiro–Wilk test prior to analysis. Independent *t*‐tests were conducted for continuous, normally distributed variables and Mann–Whitney *U* tests were performed on nonnormally distributed variables. In the case of a missing date, we imputed the day as the midpoint of the month; this occurred once. In one other case, only the year was available, therefore July 1st was the imputed date. Follow‐up time was calculated in years, from transplant to May 2025, and for deceased patients was censored at the date of death. Cases of retransplantation post‐UC diagnosis were not included in statistical analyses. A *p*‐value of <0.05 was considered statistically significant. Tumours were grouped into low‐risk (Ta G1/G2 + T1 G1/G2) or high‐risk (T1G3 + T2 + T3) as it is a superior predictor of prognosis.^21^

## 3 RESULTS

### 3.1 Patient selection

**FIGURE 1:**
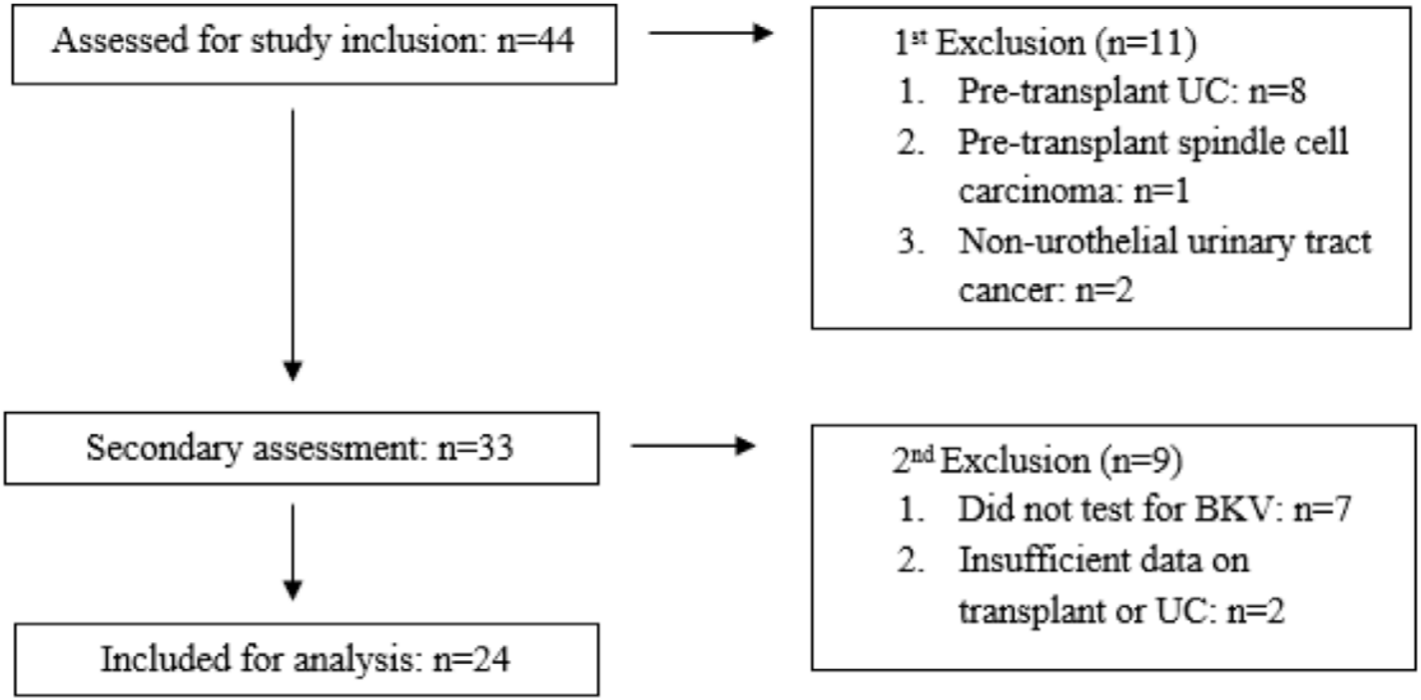
Patient selection flowchart.

Forty‐four patients were identified and after applying the exclusion criteria, 24 remained. The distribution of excluded cases is presented below (Figure 1).

### 3.2 Overview of study population

Of the 24 included patients, 11 (45.8%) had tested positive for BKV. There was a male predominance (75%), but no statistical difference between the BKV+ and BKV− cohort (*p* = 0.357). The mean follow up time post‐UC diagnosis was 7.3± 6.1 years. Mean follow‐up time post first transplant was 18.7± 12.2 years. There was approximately 1 new diagnosis per year. The median days between first transplant and UC diagnosis was 9.4 (IQR 4.31–21.72) years.

The mean age of patients at first transplant was 46.6± 18.0; 52.2% of patients underwent a living‐donor transplantation for their first transplant, and 50% of their last transplants were LDT. The mean age at UC diagnosis was 59.9± 14.6; 79% of the patients had UC involving the bladder, 17% had UC only in the allograft ureter, and 4% was isolated to the renal pelvis.

55% of the patients had a history of smoking tobacco; no statistical difference regarding BKV status (*p* = 1.000). Additionally, seven individuals underwent retransplantation; only two of these occurred pre‐UC diagnosis. No patients underwent more than 1 retransplantation.

### 3.3 Tumour characteristics

To assess the impact of BKV reactivation, we compared the stage and grade of the UC at diagnosis (Table 1). BKV+ patients had UC with a higher grade at diagnosis (*p* = 0.013); 90.9% of the tumours (*n* = 9) in patients with BKV were high grade (G3), compared to 38.5% (*n* = 5) of the BKV− patients.

**TABLE 1:** Statistical analysis of KTRs with UC stratified by BKV status.

Characteristics	Whole cohort	BKV+ cohort	BKV− cohort	*p*‐Value
	*N* = 24	*N* = 11	*N* = 13	
Male, %	75 (18)	70 (7)	84.6 (11)	0.357
Mean follow‐up post‐UC diagnosis ± SD	7.31 ± 6.10	5.74 ± 5.80	8.65 ± 6.25	0.252
Mean follow‐up time post‐transplant ± SD	18.7 ± 12.21	14.09 ± 8.69	22.62 ± 13.67	0.088
Tobacco use, %	55 (11)	50 (5)	60 (6)	1.000
Transplant factors				
Living donor for first transplant, %	52.2 (12)	45.5 (5)	58.3 (7)	0.684
Living donor for second transplant (Pre‐UC), %	47.8 (11)	36.4 (4)	58.3 (7)	0.414
Mean age at first transplant (first), ±SD	46.58 ± 17.98	54.91 ± 16.88	39.54 ± 16.29	0.034
Mean age at last transplant, ±SD	47.63 ± 17.32	56.82 ± 17.92	39.85 ± 15.99	0.025
Urothelial Carcinoma Diagnosis				
Mean age at UC diagnosis ±SD	59.92 ± 14.63	63.82 ± 17.20	56.6 ± 11.75	0.238
Median years between first transplant and UC diagnosis (IQR)	9.44 (4.31–21.72)	9.07 (4.08–10.08)	10.09 (4.52–29.79)	0.228
				Mann–Whitney *U* score: 50.00
Median years between last transplant and UC diagnosis (IQR)	8.76 (3.75–14.58)	7.15 (2.74–9.87)	10.09 (4.94–29.79)	0.150
				Mann Whitney *U* score: 46.00
Tumour stage at diagnosis				
Ta, %	41.7 (10)	18.2 (2)	61.5 (8)	0.099
T1, %	33.3 (8)	45.5 (5)	23.1 (3)	
T2/T3, %	25 (6)	36.4 (4)	15.4 (2)	
Grade at diagnosis				
G1/G2, %	37.5 (9)	9.1 (1)	61.5 (8)	0.013
G3, %	62.5 (15)	90.9 (10)	38.5 (5)	
Grouped staging				
Ta G1/G2 + T1 G1/G2	41.7 (10)	9.1 (1)	69.2 (9)	0.005
Ta/T1G3 + T2 + T3	58.3 (14)	90.9 (10)	30.8 (4)	
Risk factors				
Retransplant, %	8.3 (2)	9.1 (1)	7.7 (1)	*1.00*

We analysed the stage of the tumours at diagnosis. In the BKV+ group, 18.2% were Ta, 45.5% T1 and 36.4% T2/T3 compared to 61.5% Ta, 23.1% T1 and 15.4% T2/T3 in the BKV− group. There was no statistical significance in the stage distribution between groups (*p* = 0.099).

**FIGURE 2:**
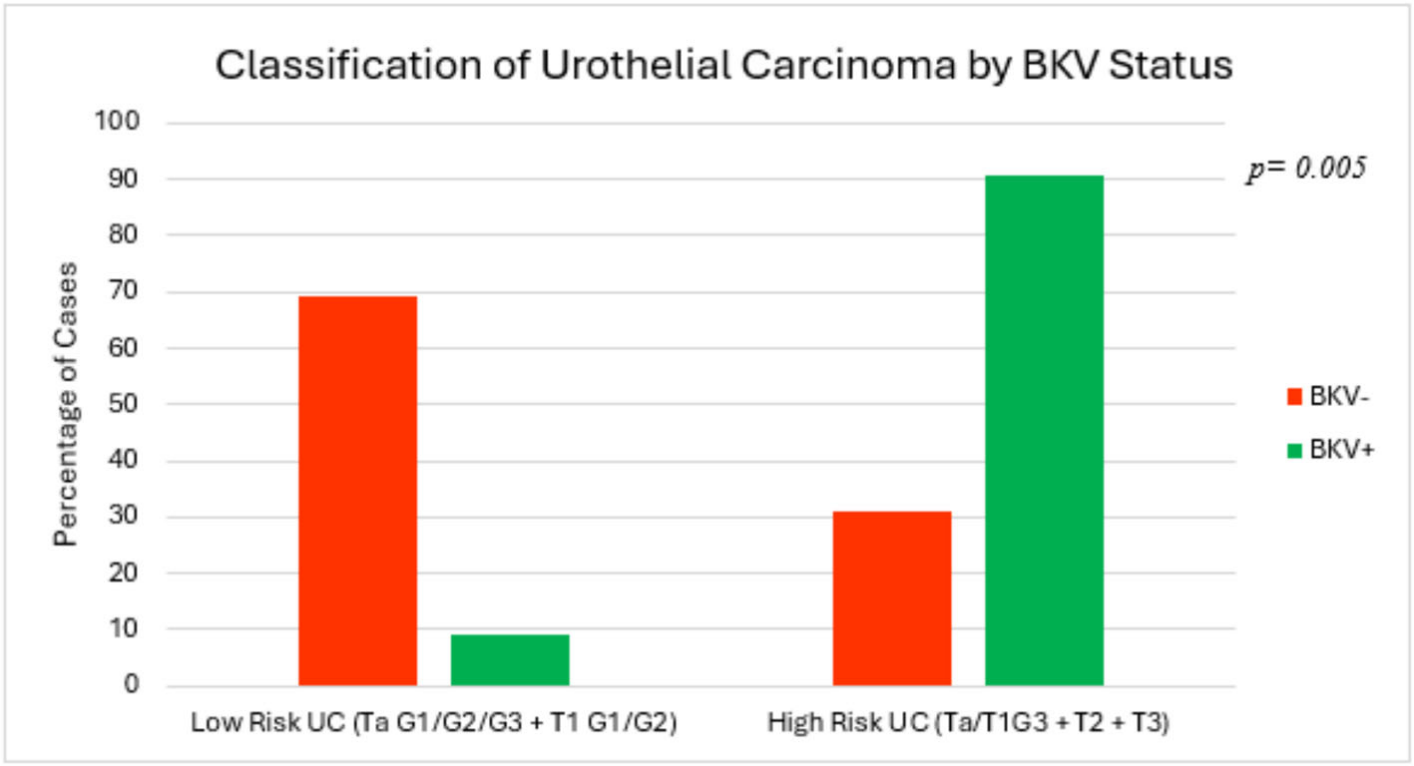
Grouped staging comparison between BKV+ and BKV− cases.

To facilitate further analysis of the effect of BKV on the oncological features of UC, we grouped the tumours into two categories: Ta G1/G2 + T1 G1/G2 and T1G3 + T2 + T3, which were low‐medium risk and high risk, respectively (Figure 2); 90.9% of patients with BKV had high‐risk UC compared to 30.8% of BKV− patients. (*p* = 0.005).

### 3.4 Onset of cancer

We compared the BKV+ and BKV− groups to assess the speed of onset of UC. The median years between first transplant and UC diagnosis for the BKV+ group was 9.1 (4.1–10.1), compared to 10.1 (4.5–29.8) years in the BKV− cohort (*p* = 0.228, Mann–Whitney *U* score = 50). This was similar for the days between the last transplant and UC diagnosis (*p* = 0.150, Mann–Whitney *U* score = 46).

The BKV+ population had their first transplant at an older age, 54.91 ± 16.9 versus 39.5 ± 16.3 (*p* = 0.034). The mean age at UC diagnosis was 63.8 ± 17.2 and 56.6 ± 11.8 years, which was similar between BKV+ and BKV− cohorts (*p* = 0.238).

### 3.5 Outcomes

**FIGURE 3:**
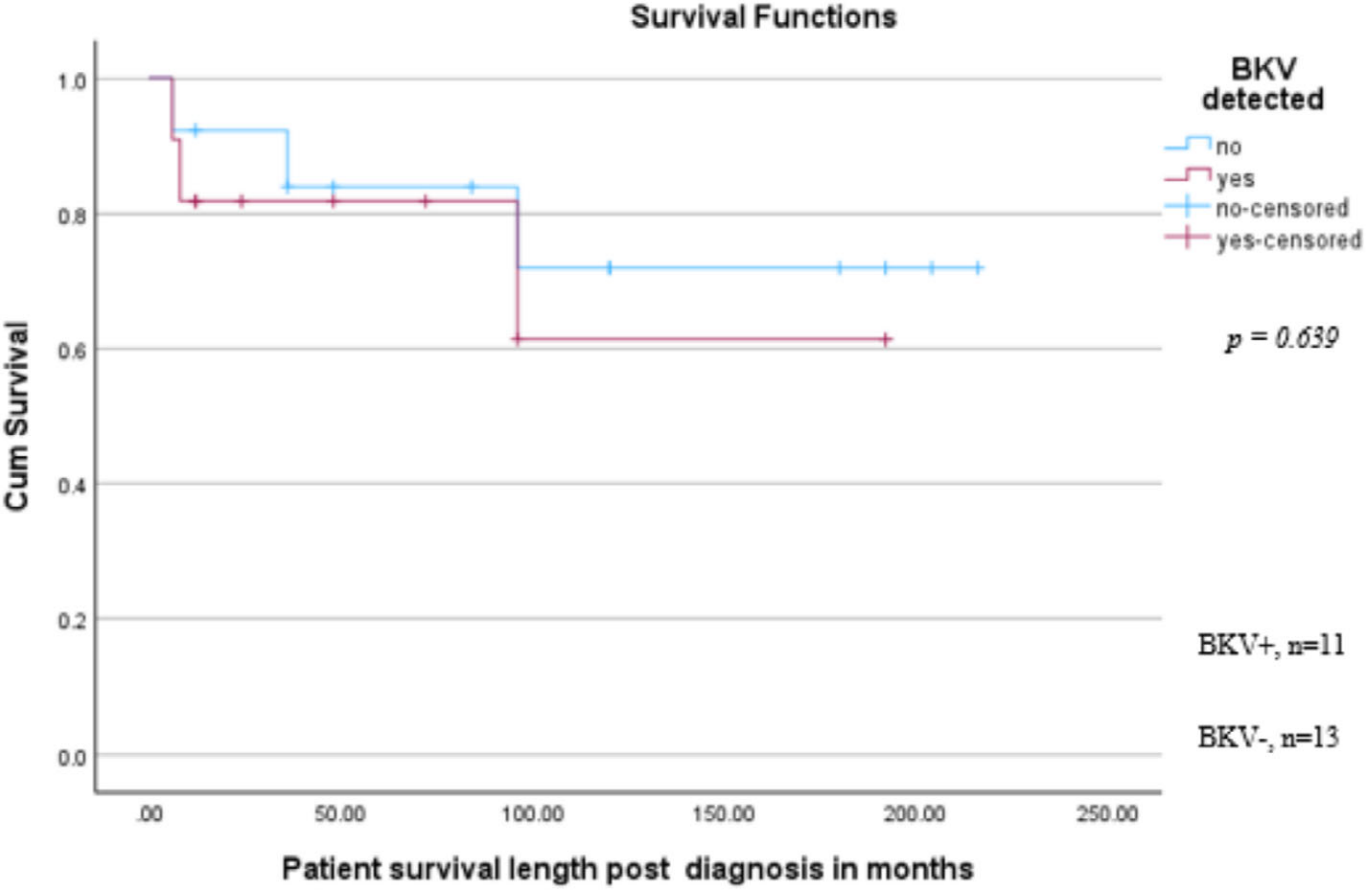
Kaplan–Meier chart of patient survival.

Six patients died during the follow‐up period: 3/11 (27.2%) in the BKV+ cohort and 3/13 (23.1%) in the BKV− cohort. A Kaplan–Meier test assessed the survival probability over time between the BKV+/BKV− patients (Figure 3). The mean survival for BKV+ patients post‐UC diagnosis was 138.7 months (95% CI: 89.2, 188.3) compared to 170.4 (95% CI: 125.6, 215.1) for those without evidence of BKV infection (*p* = 0.639).

### 3.6 SV40 antigen testing

Eighteen tumours underwent SV40 histopathological testing to assess for viral integration, allowing us to perform a subgroup analysis to explore clinical and pathological differences in SV40+ (*n* = 6) and SV40− negative (*n* = 12) UC (Table 2). Comparison of gender distribution, tobacco use, type of kidney donation and mean age at transplant resulted in no significant differences.

**TABLE 2:** Statistical analysis of KTRs with UC stratified by SV40 status.

Characteristics	Total	SV40+	SV40−	*p*‐Value
	*N* = 18	*N* = 6	*N* = 12	
Male sex, %	72.2 (13)	50 (3)	83.3(10)	0.268
Mean follow up post‐UC diagnosis in years± SD	7.32 ± 6.10	7.94 ± 6.85	7.75 ± 6.41	0.954
Mean follow up time post first transplant in years± SD	18.71 ± 12.2	16.33 ± 7.84	20.92 ± 14.95	0.496
Tobacco use, %	57.1 (8)	20 (1)	55.5 (5)	0.301
Transplant factors				
Living donor for first transplant, %	35.3 (6)	16.7 (1)	45.5 (5)	0.333
Living donor for 2nd transplant (Pre‐UC), %	29.4 (5)	16.7 (1)	36.4 (4)	0.600
Mean age at first transplant, ±SD	46.58 ± 17.99	44.83 ± 15.96	47.25 ± 20.72	0.806
Mean age at last transplant ±SD	47.63 ± 18.65	44.83 ± 15.96	49.00 ± 22.18	0.689
Urothelial carcinoma diagnosis				
Mean age at UC diagnosis ±SD	59.92 ± 14.63	53.00 ± 16.53	63.33 ± 11.74	0.143
Median years between first transplant and UC diagnosis (IQR)	9.84 (6.21–23.67)	9.44 (6.92–10.08)	10.09 (2.96–30.81)	0.616
				Mann–Whitney *U* score: 30
Median years between last transplant and UC diagnosis (IQR)	9.44 (3.64–10.68)	9.44 (6.92–10.08)	9.12 (2.69–30.81)	1.000
				Mann Whitney *U* score: 36
Tumour stage at diagnosis				
Ta, %	33.3 (6)	0	50 (6)	0.050
T1, %	33.3 (6)	66.7 (4)	16.7 (2)	
T2/T3, %	33.3 (6)	33.3 (2)	33.3 (4)	
Grade at diagnosis				
G1/G2, %	22.2 (4)	0	33.3 (4)	0.245
G3, %	77.8 (14)	100 (6)	66.7 (8)	
Grouped staging				
Ta G1/G2 + T1 G1/G2, %	27.8 (5)	0	41.7 (5)	0.114
TaG3/T1G3 + T2 + T3, %	72.2 (13)	100 (6)	58.3 (7)	
Risk factors				
Retransplant, %	5.6 (1)	0	8.3 (1)	1.00

The histology of the SV40+ and SV40− tumours was compared to assess the nature of these tumours; stage at diagnosis was statistically significant (*p* = 0.050), with all SV40+ tumours having a stage of T1 or higher, compared to only 50% of SV40− tumours. High‐grade tumours (G3) were more common in the SV40+ population (100% vs. 66.7%), but this did not reach statistical significance (*p* = 0.245). Similarly, all SV40+ were high‐risk (Ta/T1G3 + T2 + T3) compared to 58.3% in the SV40− group (*p* = 0.114).

Mean age at UC diagnosis was lower in the SV40+ group, 53.0± 16.5, compared to the SV40− group, 63.3± 11.7, although nonsignificant (*p* = 0.143). Additionally, the speed of onset was assessed by analysing years from transplant to UC diagnosis; no statistically discernible relationship was found (*p* = 0.616).

### 3.7 Allograft ureter

Five cases of UC in the allograft ureter were detected, of which three were positive for BKV, accounting for 60% of this population (Table 3). Of the BKV+ cases, two UC tested positive for the SV40 antigen, implying BK viral integration into these tumours. Given the small sample size, no formal statistical testing was performed; however, all cancers were invasive and of a high‐grade (G3). The BKV+ tumours seemed to develop at a faster rate post‐transplant, a range of 7–11 years, compared to 29–32 years in the BKV− group.

**TABLE 3:** Characteristics of patients with UC in the allograft ureter.

Case	Gender	Smoker	BKV status	SV40 testing	Histology	Years from Tx to diagnosis
1	F		−	−	T3G3	32
2	M		−	−	T3G3	29
3	M	No	+	+	T1G3	11
4	M	No	+	+	T2G3	7
5	F	Yes	+	−	T3G3	10

## 4 DISCUSSION

This study provides further evidence supporting an association between BKV infection and the formation of high‐grade, aggressive UC in the KTR population. Patients in the BKV‐positive and SV40‐positive groups had UC of a higher grade and stage at diagnosis, suggesting their tumours were more invasive and aggressive.

We found that 90.9% of BKV+ patients had high‐risk tumours compared to 30.8% of BKV− individuals, which was statistically significant (*p* = 0.005). The grade of the tumours was more impacted by BKV (*p* = 0.013) than the stage (*p* = 0.099). Similarly, Gupta et al. found that all patients with treatment for BKVN had high‐grade, invasive UC^16^. 63.6% of patients of KTRs with polyomavirus infections also had high‐grade UC^4^. Furthermore, a literature review on BKV associated UC by Iwasaki et al. found that 95.1% (*n* = 39 of 41) of tumours were of a high grade and that 53.8% (*n* = 21 of 39) had advanced‐stage disease^17^. Although there is significant heterogeneity when comparing these studies, together they provide further evidence for the aggressive, invasive nature of UC in KTRs with BKV reactivation.

Surprisingly, we found no significant correlation between the time interval between KT and UC diagnosis when comparing BKV+ and BKV− patients. Li et al. found a significantly faster onset in KTRs with BKVN compared to those without (*p* = 0.025)^15^. The same paper found that KTRs with BKVN had a younger mean age than those without (48.4 ± 13 vs. 55.7 ± 12.9, *p* = 0.038)^15^. This contrasts with the results of this study, which found no significant difference in age at diagnosis. We hypothesise that BKVN, rather than sole viruria/viremia, is more strongly associated with the earlier development of UC.

This paper found that a higher mean age at transplantation may increase the risk of BKV reactivation and, by extension, increase the risk of high‐grade UC. The mean age at first transplant was higher for the BKV+ group, 54.9 ± 16.9 vs 39.5 ± 16.3 (*p* = 0.034). However, a meta‐analysis by Demey et al. found that the effect of recipient age on BK viremia was minimal, with an OR of 1.03 (*p* = 0.016)^22^.

Luo et al. published the only retrospective cohort study comparing LTag+ and LTag−UC in the KT population, focusing specifically on UTUC (23). There was a similar rate of LTag+ tumours between our study and theirs, 33.3% versus 25.9%, respectively. We found that all SV40+ UC was high risk and of a high grade, aligning with their results, where all LTag+ UTUC was invasive. Our study found that SV40+ UC had a higher stage at diagnosis (*p* = 0.050), which was not apparent when comparing the BKV+ and BKV− patients *(p* = 0.099). KTRs with LTag‐positive UTUC had a younger age at cancer diagnosis, 48.1 ± 8.3 years, compared to those without, 54.6 ± 4.1 years (*p* = 0.013)^23^. Similarly, our patients with SV40+ tumours had a younger age at diagnosis, 53.0 ± 16.5 compared to 63.3 ± 11.7. Although this was not a significant difference (*p* = 0.143), this finding is notable as the mean age at diagnosis in BKV+ patients was higher (63.8 ± 17.2 vs 56.6 ± 11.7).

The small sample size of this study limited its statistical power, preventing us from making definitive conclusions and performing further subgroup analysis. Owing to the significant time span of our cases, with the earliest transplant occurring in 1975, not all clinical details were available, introducing a missing data bias and limiting analysis.

In the 2000s, there was an emerging awareness of the risks of BKV infection in KTRs, particularly BKVN and allograft loss. Routine BKV post‐RT screen testing was not common. The first formal UK guidelines were published in 2024.^24^ As a result, undiagnosed asymptomatic cases may have been missed in earlier cohorts because viral loads could have declined below detectable thresholds when testing was performed later. This results in the possibility of missing BKV+ cases. Spikes in BK viraemia may be sufficient to induce carcinogenic changes; therefore, misclassification of these cases impairs our statistical analysis.

BKV status was considered positive if there was evidence of BK viruria or viremia via PCR detection, or BK nephropathy at any point in the follow‐up period. This allows for cases where BKV reactivation occurred post‐UC diagnosis. There was only one case where BKV was first detected post‐UC diagnosis. Since BK reactivation occurs within the first 2 years of transplantation 95% of the time, we included this case.^25^

Despite the limitations, this study provides valuable insight into the relationship between BKV and UC. Our paper identified 11 BKV+ patients, providing a larger BKV+ cohort than many earlier reports, thereby strengthening the available evidence. The long follow‐up time exceeded previous observation windows, allowing for robust assessment of UC formation over time.

Among our patients, five presented with UC in the allograft ureter; all tumours were high‐grade. This aligns with previous research, stating that UC of the transplant ureter frequently presents as aggressive, high‐grade disease^26^; 60% of these patients had tested positive for BKV. Notably, this subgroup appeared to have a much faster rate of onset of UC. Enhanced donor screening, vigilant post‐transplant surveillance and additional research into BKV dynamics in the renal allograft are essential to mitigate this risk.

Current management guidelines highlight the importance of early and regular screening for BKV, due to its asymptomatic nature in the early stages of reactivation. The British Transplant Society recommends plasma BKV‐DNAaemia PCR diagnostic screening every 3 months for the first 2 years and annually in years 3–5 post‐transplant, as a minimum.^24^ Subsequently, the aim remains early reductions in immunosuppressive regimens, attenuating the risk of BKVN.^27^ Given the emerging evidence surrounding the increased risk of high‐grade UC associated with BKV infection, a screening protocol in this population is warranted. Post‐BKVdetection, periodic urine cytology or cystoscopy may be effective at identifying developing UC at an earlier stage. Additionally, analysis of longitudinal viral monitoring is required to understand the dynamics of viral load and UC development.^24^ Research should explore if BKV viraemia spikes can induce carcinogenesis, even if followed by remission of the BKV infection. There are no antiviral or prophylactic therapies for BKV currently; however, vaccines for BKV are in development.^28^

## AUTHOR CONTRIBUTIONS

*Research conception and design*: Jonathon Olsburgh, Rhana Zakri, Luis Ribeiro, Noah Beetge, Catherine Horsfield. *Statistical analysis*: Jonathon Olsburgh, Rhana Zakri, Luis Ribeiro, Noah Beetge. *Drafting of the manuscript*: Jonathon Olsburgh, Rhana Zakri, Luis Ribeiro, Noah Beetge. *Critical revision of the manuscript*: Jonathon Olsburgh, Rhana Zakri, Luis Ribeiro, Noah Beetge. *Approval of the final manuscript*: Jonathon Olsburgh, Rhana Zakri, Luis Ribeiro, Noah Beetge.

## CONFLICT OF INTEREST STATEMENT

The authors declare no conflicts of interest.
